# Participatory Design of a Mobile App to Safeguard Mental Resilience in the Context of Drug Use in Young Adults: Multi-Method Study

**DOI:** 10.2196/34477

**Published:** 2022-02-25

**Authors:** Ofri Ben-Yehuda, Efrat Dreazen, Danny Koren, Mor Peleg

**Affiliations:** 1 Department of Psychology University of Haifa Haifa Israel; 2 Department of Information Systems University of Haifa Haifa Israel

**Keywords:** mobile health, mHealth, eHealth, telehealth, mental health, mental resilience, participatory design, mobile phone

## Abstract

**Background:**

Existing mental health apps are largely not aimed at generally healthy young people who may be experimenting with addictive substances and mind-altering experiences.

**Objective:**

The aim of this study is to examine the interest and expectations of young people regarding a proposed smartphone app designed to help protect and promote mental health and resilience in the face of risks associated with substance use.

**Methods:**

The study was based on agile system development and had 3 empirical substudies. Our feasibility study (study 1) included an anonymous questionnaire that examined the potential interest of young people in this type of app. It was answered by 339 Israelis aged 18-30 years. The second part of the feasibility study was a pilot study with 1.2% (4/339) of the people who answered the questionnaire and expressed interest in participating in a focus group. They tested and refined the elements planned for the focus groups. Study 2 was a participatory design study involving 7 focus groups of 5 to 7 participants each (young people aged 18-35 years, n=38). Persona development, open discussion, and a Technology Acceptance Model questionnaire were used to elicit user expectations and requirements for the app and to understand the perceived usefulness and usability of the proposed features. Study 3 comprised in-depth interviews with experts in the field of youth mental health and drug use to enlist their professional opinion regarding the value of such an app and recommendations about the features it should include.

**Results:**

The mock-up for the proposed app had five key features: personalized assessment of risk for a drug-associated mental crisis, support for self-monitoring, useful information (eg, warning signs and first-aid guidelines), resilience-building exercises, and a support center. Participants rated highly the usefulness of all 5 main features and 96% (24/25) of the specific features we proposed within those main categories. The participants also suggested additional features as well as a new user persona we had not considered: the parents or family members of the young person. The focus groups rated highly the perceived usability of the app. Most of the experts saw value in all the main features and suggested specific knowledge sources for the app’s content. Finally, participants of both the feasibility study and the participatory design study expressed moderate to high interest in using the app for self-help and high interest in using the app to help friends.

**Conclusions:**

The findings provide preliminary encouraging support for the 5 main features suggested by the research team and reinforce recommendations for mobile health apps found in the literature. The findings emphasize the insight that this kind of app should be designed primarily for use by individuals seeking to help others.

## Introduction

### Background

Adolescence and young adulthood are characterized by major changes in all areas of development: social, emotional, physical, and cognitive [1]. The important changes during this period generate instability and uncertainty and a significant mental health risk [2]. Studies indicate that approximately 75% of the psychological morbidity experienced throughout life erupts during early adulthood [3,4].

Moreover, mental disorders that develop during this age often persist, disrupting the capacity of young people to fulfill their potential [5], limiting access to mental and physical health care [6], and exposing them to poor educational and reduced occupational opportunities [7], stigma, social isolation, discrimination, and violation of human rights [8], as well as higher morbidity and mortality risks (including suicide) than the general population, translating into a striking 10-20 years’ reduction in life expectancy [9,10].

The risk factors for developing emotional problems in adolescents and young adults include the use of drugs or other addictive substances, the pursuit of stimulating and spiritual experiences, dangerous behavior and delinquency, unsafe sex, and exposure to trauma [11]. Our main interest here is in the first 2 of these, primarily the use of addictive drugs.

In Israel, as elsewhere, recent years have seen a rise in the number of young people engaging in the use of drugs, mainly cannabis, and in mind-altering techniques, including spiritual and religious practices [12]. Both are particularly common in the context of long-term backpacking—a social phenomenon in which young people travel to exotic (for them) locales, often rich in natural wonders, for periods lasting a few months to a year or more. Long-term backpacking is prevalent among young Israelis, approximately 50,000 of whom set out on such trips every year [13]. Estimates of drug use among Israeli backpackers range from 50% to 90% [13,14]. Each year, approximately 2000 Israeli backpackers are negatively affected by drugs during their trip. Of these, approximately 600 are forced to return to Israel for mental and physical treatment, and dozens require immediate rescue [14].

Alongside their being at increased risk for mental problems, adolescents and young adults still retain a certain degree of physical and psychological plasticity, which facilitates early preventive interventions [15]. Yet, despite the high potential of early identification and intervention to improve young people’s physical and mental health, many of those at high risk fail to take advantage of the various professional services on offer [16]. This failure may have many causes, ranging from a lack of information about the risks to psychological inhibitions stemming from social stigma regarding distress at this age [17]. In accordance with this latter explanation, several studies have observed that young adults are more likely to ask for help for their friends than for themselves [18,19]. Likewise, there is evidence that young people are more willing to seek out information when it is offered over the internet [20]. This pattern might reflect both the convenience of accessing information on the web [19] and the fact that seeking help on the web allows the seeker to remain anonymous [21]. This, in turn, may diminish the stigma associated with a sense of being isolated and different, strengthening young people’s willingness to access forms of support that may improve their sense of meaning and self-worth [22,23].

For these and other reasons, over the past 2 decades, there has been a substantial rise in the number of mobile health (mHealth) apps focusing on mental health [24-31]. A 2017 survey reported that >10,000 mental health and wellness apps are available for download [32]. Therefore, we first searched the web for an app with a purpose similar to ours—to promote mental health and prevent drug-induced psychosis among young adults. Although we did not find one that meets these requirements, we found many apps that are each related to some of the different mental health and wellness issues. Specifically, we searched for studies related to apps designed to prevent or treat early psychosis, support an individual in mental distress (experiencing anxiety, depression, or posttraumatic stress disorder symptoms), build mental resilience, and minimize the harms of substance use. We focused on apps targeted at young people and that were demonstrated as effective. We found that the main features offered by such apps include information and psychoeducation, tools for developing mental resilience, risk or symptoms assessment, and support for self-monitoring and independent acquisition of therapeutic techniques (Table 1). Some apps are even intended to replace traditional face-to-face therapies, such as those based on cognitive behavioral therapy (CBT) [33]. We also found these features, excluding useful information related to drugs, in an app that is intended to develop resilience in youth without mental disorder diagnoses (Table 1). Apps that focus on harm reduction of drug use (Table 1) mostly offer educational information and contact details for support services but do not offer personalized risk assessment or resilience-building methods. However, the effectiveness of these 2 apps was not evaluated in research. We found supporting studies on apps designed to manage substance abuse disorder, but they do not meet our goals.

**Table 1:** Features of sample mental health apps and apps that offer drug education and support.

App name	Target end users	Risk assessment	Self-monitoring	Useful information	Mental resilience development	Support center	Reference
Joy Pop	Youth	✓	✓		✓	✓	[25]
RobinZ	Young people at risk for psychosis	✓	✓	✓	✓	✓	[26]
ThinkApp	Youth after first episode of psychosis	✓		✓	✓	✓	[27]
Actissist	Early psychosis relapse	✓	✓	✓	✓	✓	[28]
PTSD^a^ coach	PTSD	✓		✓	✓		[29]
SAM^b^	Anxiety disorders		✓		✓	✓	[30]
Optimism	Mood disorders		✓		✓		[31]
MindZone	Drug users			✓		✓	[34]
TripSit	Drug users			✓		✓	[35]

^a^PTSD: posttraumatic stress disorder.

^b^SAM: Self-help for Anxiety Management.

Another shortcoming of existing apps designed to promote mental health is that, despite the extensive research conducted in this area, most of these apps (approximately 98%) have not been accompanied by evaluation studies [24,36]. However, meta-analysis studies found some support for effectiveness in health promotion and management of various emotional problems of young people [37,38].

After examining the existing literature, a central question that remained was whether young people would be disposed to use an app that could protect their mental health (ie, lower the risk of mental breakdowns associated with drug use and engagement in mind-altering spiritual experiences) and whether they would use it for a relatively long period of time (eg, for the entire duration of their backpacking trips, which usually span several months).

### Proposed App

In this research, we used agile software development methods [39], including participatory design methods [40], to derive “empathetic solutions that are more desirable to target populations” [41] and to evaluate designs for them, which also draw from behavioral theories and existing apps. The solution that we derived, based on the aforementioned principles, is a smartphone app designed to help young people cope with challenges posed by young adulthood, with an emphasis on substance use, by delivering personalized information, guidance, and support. The proposed SafeGuard app is envisioned as offering five high-level features, similar to those reported in the literature and summarized in Table 1: risk assessment (personalized assessment of both generalized and situational risk for a drug-induced mental crisis), support for self-monitoring (eg, a status assessment and daily recommendations), personalized information related to drug use and treatment of worrying symptoms, exercises and coping strategies (including CBT) to increase mental resilience, and a support center offering quick access to support and help. The main goals of this study are to address the aforementioned open questions by (1) examining whether young people find the idea of the app beneficial as well as the potential interest of young people in using such an app and (2) assessing the expectations of both young people themselves and health care professionals regarding the contents of the app based on the 5 high-level feature categories. Our research objectives are as follows:

- Objective 1a: To obtain young people’s views about the proposed SafeGuard app, specifically whether they view it as likely to be beneficial. This was operationalized as (1) perceiving recreational drug use as widespread, (2) perceiving existing information about the risks of drug use as insufficient, and (3) perceiving their friends as likely to use the app. This third condition is based on a large body of literature showing that young adults are more likely to ask for help for their friends than for themselves [18,19].
- Objective 1b: To assess whether young people express interest in using the proposed app, whether for self-help or for the benefit of their friends. This was reflected by reported intentions to download and use the app, and to recommend it to their friends.
- Objective 2: To elicit requirements for the app from young people.
- Objective 3: To obtain experts’ opinions about the perceived usefulness of the proposed app and enlist their requirements as well as their opinions about the proposed features gathered according to the first stages of the research: risk assessment, self-monitoring, useful information, resilience development, and a support center.
- Objective 4: To acquire specific knowledge sources that could potentially be used to implement these features.

Objective 1a served as a feasibility assessment and was tested with a large cohort of young adult participants (n=339; study 1). Objective 1b and objective 2 were addressed through qualitative research with participants (n=38) from the same population as study 1, using a participatory design methodology with mock-ups of the proposed app (study 2). Objective 3 and Objective 4 were addressed by means of interviews with health care professionals (N=10) as part of the app development (study 3).

## Methods

### Overview

The study is based on agile system development—a set of practices in which requirements are gathered and the software is developed, tested, and improved in an iterative process [39]. We first conducted our feasibility study (study 1). We then relied on our knowledge of the literature and the results of study 1 to create initial mock-ups for the app. Next, we used participatory design methods to allow participants of the focus groups from the population of intended users to develop personas and requirements for the app and to evaluate the initial mock-ups through an anonymous questionnaire (study 2). The mock-ups were also evaluated by experts (study 3). The combined methods form triangulation (cross-referencing of data from at least three different independent sources of information, such as observation and interviews) [42,43] and thus help us overcome the limitations of qualitative research.

### Ethics Approval

Ethics approval was obtained from the ethics committee of the University of Haifa (#160/19) on May 9, 2019.

### Study 1: Feasibility

#### Participants

Our intention was to recruit 500 respondents to have a representative sample of the 50,000 young Israelis who set out on backpacking treks each year [14]. Participants were recruited using the snowball method through announcements posted in internet discussion groups and forums, representing a convenience sample. The announcements included a short explanation of the research objectives and the option to choose to participate in a short survey. In addition, the announcements indicated that participation in the study would enable participants to enter a lottery for a large trekkers’ backpack. Participants comprised 339 Israelis aged 18-30 years who were not currently serving in the military. The participants were accepted into the study between May 19, 2019, and August 14, 2019. All participants signed an electronic informed consent form.

#### Procedure and Measures

The young people who agreed to participate in the feasibility study were asked to complete a short anonymous questionnaire. Basic sociodemographic background information (age, gender, religious background, occupational status, etc) was collected to ensure the representativeness of the study sample. The main measures comprised three 5-point Likert scale questions. The first two assessed participants’ perceptions regarding the extent of drug use among young people and the availability of information on the topic—specifically, “My impression is that use of recreational drugs is a common phenomenon” and “My impression is that young people do not have enough accessible information about the risks associated with drug use” (1=Do not agree at all, 5=Agree completely). The third introduced the main features of the app as described previously and asked, “To what extent do you think your friends will use the proposed app?” (1=Not at all, 5=Daily). At the end of the questionnaire, participants interested in joining a focus group were invited to notify the study organizers (see the *Study 2* section). Of the 339 participants, 4 (1.2%) took part in a 2-hour pilot in which we tested and refined the elements planned for the focus groups.

#### Data Analysis

Descriptive statistics means and SDs were calculated using SPSS software (version 25.0; IBM Corp). Potential associations between intention to use the app (operationalized as an agreement that friends would use the app) and demographic characteristics were tested using Spearman correlations and analysis of variance (ANOVA).

### Study 2: Participatory Design

#### Overview

**Figure 1:**
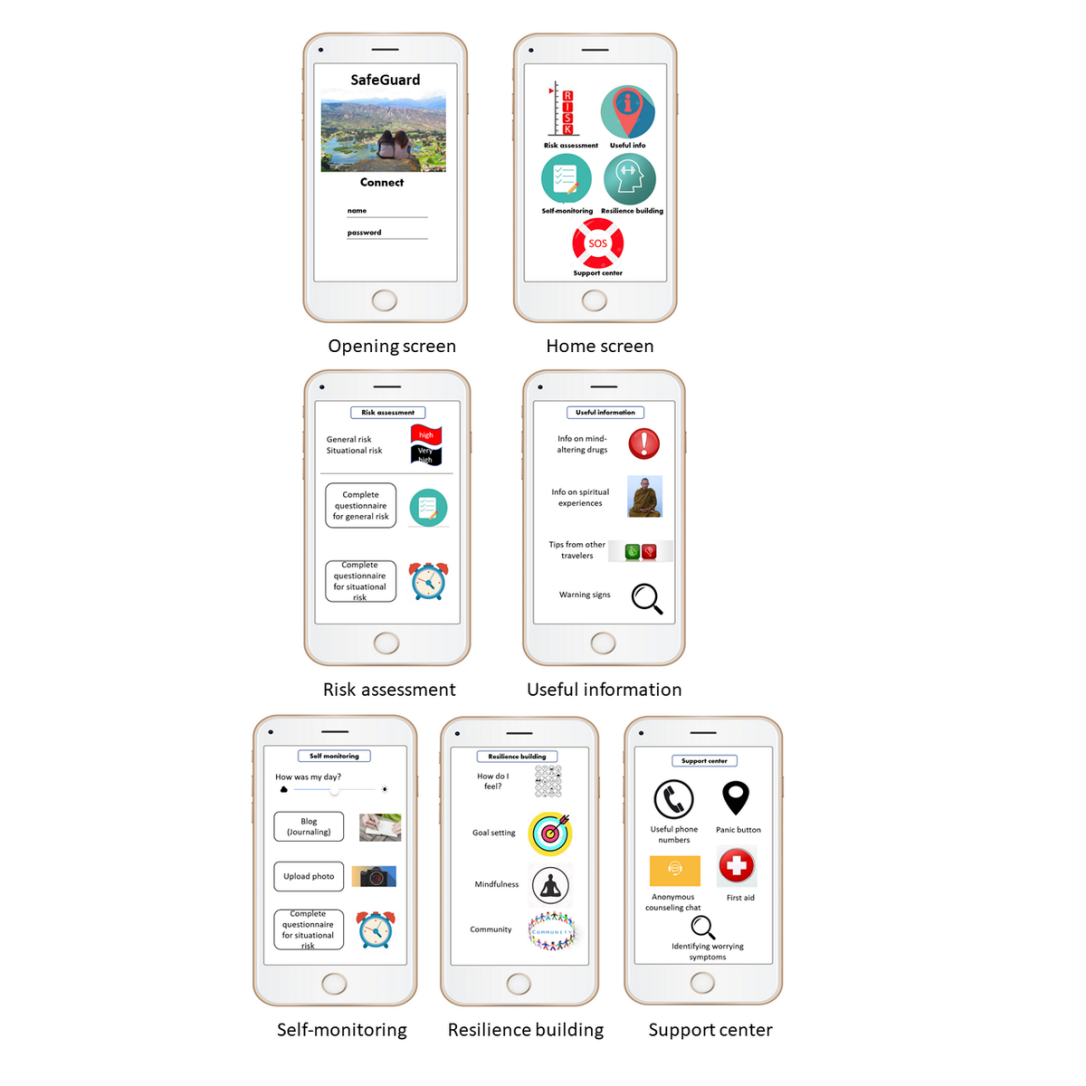
User interface mock-ups of the app’s screens.

As mentioned previously, agile software development [39] is a methodology in which requirements are gathered and the software is developed, tested, and further improved in an iterative process. The team that develops the software includes developers and potential users, an approach that is also known as participatory design [40]. We applied the first 2 phases of the Integrate, Design, Assess, and Share (IDEAS) framework of agile strategies, which draws on evidence-based theories to develop more effective digital interventions to change health behavior [41,44]. In the *Integrate* phase, we integrated insights from users through qualitative research, including focus groups and questionnaires, to understand the target population and their needs. The focus of our qualitative study was on understanding the drug-related behavior of young people and their willingness to make use of, and benefit from, an app that could help them develop mental resilience and lower their risk of mental breakdowns, which is increased by substance abuse. To ground the proposed app in behavioral theories, we drew for this study on psychological theories of resilience and related theory-based behavioral strategies, including CBT [45-48]. In the *design* phase of IDEAS, our multidisciplinary coauthors’ team carried out the ideation phase by brainstorming creative strategies for translating these theories into requirements for specific app features for strengthening executive functions (planning, awareness, and flexibility) and fostering interpersonal communication. The features included web-based groups (chat style) of the app users, setting personal goals, self-reporting and monitoring, and mindfulness techniques. We also drew requirements from our review of the literature and of related apps, arriving at 5 high-level feature categories of the app, described in the *Introduction* section. On the basis of the extension of IDEAS in the study by Peleg et al [44], we translated the theory-based feature into user interface (UI) mock-ups (Figure 1) that partition the features into a hierarchy of app screens. The mock-ups were evaluated in studies 2 and 3.

Study 2 used a participatory design methodology. Participatory design involves potential users in eliciting requirements (the first stage of agile development) and evaluating their implementation [49]. Participatory design has often been used to develop mHealth apps [50-53]. Different participatory design techniques exist [49]. We used focus groups, persona development, user feedback, and a questionnaire, as detailed in the next sections.

#### Participants

Participants in this study were young Israelis (n=38) aged 18-30 years who were not currently serving in the military. Participants were recruited in two main ways: (1) through direct invitations to participants in study 1 and (2) through advertisements posted in 15 web-based discussion groups and forums. All participants provided informed consent. They were divided into 7 focus groups of 5 to 7 participants each.

#### Procedure and Measures

Each focus group met for a similar 2-hour session held in either Tel Aviv or Haifa (Israel’s main central city and northern city, respectively). The sessions had 3 components, based on standard participatory design practices [49]. First, participants suggested hypothetical personas, characterizing prototypical users and formulating *stories* about situations where they would use the proposed app and their expectations from it. Next, the research team presented the aforementioned UI mock-ups and solicited feedback regarding the proposed features (and any features deemed by participants to be missing) through open discussion. Discussions regarding the personas and mock-ups were audio recorded and later transcribed for qualitative analysis.

Finally, participants responded anonymously to a written questionnaire (adapted from the Technology Acceptance Model questionnaire [54]) assessing the perceived usefulness of specific features of the app and intentions to use the fully functioning app in the future. In part 1 of the questionnaire (25 items), participants were asked to rate the importance of each feature, whereas in part 2 (11 items), they were asked to rate the usefulness of the app and their intentions to use it themselves or to help friends (see details in the *Study 2*: *Participatory Design* section under *Results*). The results were stored in the research software without linking the details of the participants in the focus groups and their email addresses to the questionnaire data. In addition, the focus group protocol was securely maintained by the research team and was not published or transmitted in any way.

#### Data Analysis

Objective 1b: Intentions to download and use the app for self-help or for the benefit of their friends and to recommend it to friends were examined through descriptive statistics, including means, SDs, and distribution of the ratings. In addition, we tested whether and to what extent participants intended to use the app themselves or to help friends by subjecting the relevant questionnaire responses to a 2-tailed dependent means *t* test (see the *Results* section). Finally, associations between demographic and drug use characteristics (age, gender, and different types of drug exposure) and intentions to use the app were tested using ANOVA followed by a multivariate linear regression model.

### Study 3: Expert Interviews

Interviews are an efficient and focused method of gathering experts’ opinions [55] and are used in the IDEAS [41] framework that we followed “to gain a deeper understanding of the selected target population and their needs.”

#### Participants and Procedure

We contacted 12 professionals in fields relevant to the study; of the 12, 7 (58%) were identified through a web-based search for leading professionals in the field of risks to youths’ mental health and resiliency in general and due to drug use in particular. Of these 7, 2 (29%) were contacted in earlier phases of the research. Of the 12 professionals, 5 (42%) were recommended by other interviewees. The experts were emailed a brief description of the study, and those who responded were then sent more detailed information. Of the 12 experts, 10 (83%) agreed to be interviewed, representing the following fields: substance abuse prevention, early identification and intervention with young adults at risk for mental health crises due to drug use, search and rescue specialists, and resilience development experts. Of the 10 experts, 8 (80%) were interviewed individually and 2 (20%), who work with *Safe Shore*, a psychedelic harm reduction, education, and peer support project, were interviewed together. All interviews were audio recorded and transcribed for later analysis. All the interviewed experts provided informed consent.

#### Data Analysis

Of the 10 experts, 1 (10%) agreed to be interviewed only on the topic of resilience development. For the other experts, we used semistructured interviews in which we presented the mock-ups and solicited feedback about the different features while collecting recommendations for specific content. We conducted directed content analyses [56] to assess perceived usefulness, impediments, and general impressions of the app. We used this method [56] also to reveal commonalities and differences among the opinions and suggestions of the experts regarding specific app features. The literature sources suggested by the experts were analyzed to extract content for the app’s features. 

## Results

### Study 1: Feasibility

#### Sociodemographic Characteristics of the Sample

The demographic characteristics of the feasibility study participants are shown in Table 2. Except for the overrepresentation of women, the sample was representative of the population of nonultraorthodox youth in Israel in terms of basic socioeconomic characteristics, including age, occupational status, religion, and religious orientation, based on population totals from the Organization for Economic Cooperation and Development [57] and the Israel Central Bureau of Statistics [58,59]. According to these sources, 50.9% of the adult population in Israel hold an undergraduate degree [57], 75% are Jewish [58], and 45% have a secular orientation [59].

**Table 2:** Sociodemographic characteristics of the sample in study 1 (N=339).

Characteristics		Values
Age (years), mean (SD; range)		24.3 (2.7; 18-30)
**Gender, n (%)**		
	Male	130 (38.3)
	Female	209 (61.7)
**Occupational status, n (%)**		
	Student	170 (50.1)
	Full-time job	83 (24.5)
	Before or during backpacking trip	50 (14.7)
	Premilitary	6 (1.8)
	Other	30 (8.8)
**Religion, n (%)**		
	Jewish	288 (85)
	Atheist	25 (7.4)
	Christian	10 (2.9)
	Druze	6 (1.8)
	Muslim	3 (0.9)
	Other	7 (2.1)
**Religious orientation, n (%)**		
	Secular	228 (67.3)
	Traditional	64 (18.9)
	Orthodox	25 (7.4)
	Other	22 (6.5)

#### Questionnaire Results

Table 3 presents the distribution and means of responses to 2 of the 3 main questions of the feasibility study. Most participants agreed strongly or completely that the use of recreational drugs is a common phenomenon (Q1) and felt that there is already enough educational information available regarding risks associated with drug use (Q2). As for Q3, the modal response regarding the frequency with which their friends would use an app of this kind was *sometimes* (141/339, 41.6%). Importantly, only 7.9% (27/339) of the participants responded that their friends would not use an app of this kind at all. (97/339, 28.6% indicated that they would seldom use the app, 67/339, 19.8% indicated that they would use the app often, and only 7/339, 2% said they would use it daily. A 1-way ANOVA revealed no significant differences based on gender, occupational status, or religious orientation regarding perceptions about the prevalence of drug use among young people, the accessibility of information about its risks, and friends’ intention to use the proposed app.

Table 4 presents the intercorrelations among the 3 survey questions, as well as between each question and age. As can be seen, perceptions about the accessibility of information were correlated with perceptions of friends’ intentions to use the proposed app (*P*<.001): the more they thought that there was insufficient information about the risks associated with drug use, the more frequently they thought that their friends would use the app. There was no significant correlation between age and responses to any of the 3 questions.

**Table 3:** Distribution of replies to the feasibility study questions Q1 and Q2 (N=339).

	Value, mean (SD)	1: Do not agree at all, n (%)	2: Somewhat agree, n (%)	3: Agree, n (%)	4: Strongly agree, n (%)	5: Completely agree, n (%)
Q1: My impression is that the use of recreational drugs is a common phenomenon	4.1 (0.9)	4 (1.2)	14 (4.1)	56 (16.5)	145 (42.8)	120 (35.4)
Q2: My impression is that young people do not have enough accessible information about the risks associated with drug use	2.8 (1.0)	33 (9.7)	105 (31)	120 (35.4)	59 (17.4)	22 (6.5)

**Table 4:** Matrix of Spearman correlations and 2-tailed *P* values of age and answers to the three study measures.

Variable name		Age	Q1^a^	Q2^b^	Q3^c^
**Age**					
	*r*	1.00	—^d^	—	—
	*P* value	<.001	—	—	—
**Q1**					
	*r*	0.051	1.00	—	—
	*P* value	.35	<.001	—	—
**Q2**					
	*r*	0.001	0.033	1.00	—
	*P* value	.99	.54	<.001	—
**Q3**					
	*r*	–0.094	0.033	0.255	1.00
	*P* value	.09	.54	<.001	<.001

^a^Q1: My impression is that the use of recreational drugs is a common phenomenon.

^b^Q2: My impression is that young people do not have enough accessible information about the risks associated with drug use.

^c^Q3: To what extent do you think your friends will use the proposed app?

^d^Not applicable.

### Study 2: Participatory Design

#### Overview

Figure 1 shows the mock-up screens that were developed by our team and shown to participants of the focus groups (study 2) and the expert interviewees (study 3). As can be seen, calculated risk is visualized through red and black flags (high risk and very high risk, respectively), following the analogy of warning flags used at Israeli beaches to indicate safety conditions for bathing. As a nonnegligible level of risk for a mental breakdown after drug use always exists, the mock-up does not include a white flag (used on beaches for the lowest level of risk). The actual questionnaires that will be used to assess personal levels of risk [60] were not shown in the mock-up.

#### Sociodemographic Characteristics of the Sample

The sample for this study (including the pilot group and focus group participants) was demographically similar to the sample for study 1. The mean age of the pilot group participants was 25.8 (SD 4.4) years, and 55% (21/38) were women.

#### Development of Hypothetical Personas

Hypothetical personas were developed to help formulate the potential user population for the app and the requirements of each user. The participants envisioned 4 hypothetical personas, developed based on their own experiences. Multimedia Appendix 1 presents an evidence trace table containing quotes from the participants describing the personas and the contexts in which they might use the app.

The first persona represented young people using the app for self-help purposes while on a trip abroad. Young people who match this hypothetical persona are in a phase of life marked by self-exploration and working out their own identity as they enter adulthood. They are interested in new and exciting experiences, including recreational drug use. At the same time, they are presumed to be interested in guidance and direction that might give them some perspective before they embark on new experiences.

The second persona represented young people who had been exposed to mental health challenges or mental illness in their family and who want to act responsibly and keep their eyes open while traveling alone or trying new and risky experiences, including drugs or spiritual practices.

The third persona represented parents and family members who have concerns about the well-being of a child or sibling. The proposed app might be able to help these family members approach the young person in a supportive and nonthreatening way, while also helping them cope with their own concerns and distress.

The fourth persona represented a friend in distress. Many participants believed that young people tend to ignore, or be in denial about, their own problems. Concerned individuals might be able to use the app to help friends observed to be in a troubled mental state.

#### Requirements and Desired Features of the Envisioned App

Table 5 presents the focus groups’ ratings for part 1 of the Technology Acceptance Model–based questionnaire in which participants were asked to rate the importance of various features of the app on a scale from 1 (not important at all) to 5 (necessary). As seen in Table 5, potential users (participants of the focus groups) rated as highly important 96% (24/25) of the features we introduced at the prototyping stage. The feature that they did not rate highly was the ability to upload images as part of the self-monitoring component.

**Table 5:** Means and SDs for responses on the importance of app features (part 1 of the Technology Acceptance Model–based questionnaire)^a^.

Feature and details			Value, mean (SD)
**Risk assessment**			
	The app should include a risk-assessment feature	4.13 (1.04)	
**Self-monitoring**			
	The app should include a self-monitoring feature	4.13 (0.85)	
	Image upload^b^	2.24 (1.22)	
	Icon selection to represent one’s current mood	3.74 (1.03)	
	Personal blog and daily summary	3.38 (1.42)	
	Status assessment and daily recommendations	4.29 (0.84)	
**Useful information**			
	The app should include a useful information feature	4.47 (0.71)	
	Health and mental risks related to drug use	4.34 (0.79)	
	Health and mental risks related to spiritual experiences	4.16 (0.89)	
	Effects and side effects of different types of drugs and combinations	4.66 (0.65)	
	Red flags for developing mental distress that require immediate attention	4.70 (0.45)	
	Tips for avoiding or reducing risk	4.47 (0.84)	
	Location-dependent warnings about sources and unsafe places to consume drugs	4.37 (1.1)	
**Resilience development**			
	The app should include a resilience-development feature	4.24 (0.70)	
	Strengthening self-awareness	3.81 (1.02)	
	Strengthening executive functions	3.76 (1.16)	
	Mindfulness and relaxation techniques	3.66 (1.07)	
	Interpersonal communication	3.92 (1.29)	
**Support center**			
	The app should include a support center feature	4.74 (0.43)	
	Distress button that sends a message to the insurance company	4.39 (0.80)	
	Distress button that sends a message to people you have listed	4.58 (0.54)	
	List of useful phone and location assistance centers	4.70 (0.72)	
	Guidelines for identifying signs of distress	4.51 (0.67)	
	First-aid guidelines to help a person presenting signs of distress	4.42 (0.95)	
**Anonymity**			
	It is important for me to stay anonymous while using the app	4.05 (1.08)	

^a^Participants answered all questions on a 5-point Likert scale where 1=not important at all, 2=not important, 3=neutral, 4=very important, and 5=necessary.

^b^The only app feature not rated highly by the participants.

The open discussion portion of the focus group sessions allowed participants to propose new features beyond those on our list. Altogether, 13 new features were suggested, of which 2 (15%) were raised by at least 10 different participants. The first was providing tips for parents and friends on ways to communicate concerns regarding risky drug use through supportive dialogue (12/38, 32%), and the second was a feature that would enable sharing one’s own experience or reading about others’ experiences of experimenting with drugs (10/38, 26%). In addition, many users suggested alternative ways to present various features proposed by the team. For example, participants proposed various ways for users to report on their current mood beyond the use of icons (eg, *How Do I Feel?*). The participants also suggested incorporating reminders to engage in various activities on the app, such as resilience activities or filling out a situational risk–assessment questionnaire.

Table 6 presents the means, SDs, and frequency distributions for responses to part 2 of the questionnaire. The first 2 sections of part 2 deal with the perceived usability and usefulness of the app. As can be seen, participants overwhelmingly agreed that the app would be easy to use, and most agreed or strongly agreed with 6 statements reflecting the perceived usefulness of the app. The final section of part 2 contains 3 items reflecting intentions to use the app. Regarding objective 1b, responses to the first 3 intention measures suggest that young people would indeed be willing to use the app, either for self-help or to help their friends. Specifically, 53% (20/38) of the participants agreed or strongly agreed that they would download the app for personal use (mean 3.58, SD 0.98), whereas 76% (29/38) agreed or strongly agreed that they would download the app to help others (mean 4.21, SD 0.80) and 92% (35/38) agreed or strongly agreed that they would recommend the app to a friend (mean 4.29, SD 0.60).

Regarding the frequency of use, 50% (19/38) of the participants responded that they would use the app *sometimes* (13/19, 68%) or *often* (6/19, 32%; mean 2.55, SD 0.87), although none of the users intended to use it daily and 4/38, 11% said they would not use it at all. Interestingly, almost as many participants said that they would *seldom* (15/38, 39%) use the app for their own personal needs as responded either *sometimes* (13/38, 34%) or *often* (6/38, 16%). This finding is in keeping with the fact that the proportion of respondents saying that they would use the app to help others was significantly larger than the proportion saying that they would use it themselves (t_32_=–3.125*;* *P=*.004). Both these findings support our operationalization of the feasibility study (Objective 1a) based on the idea that young people are more likely to seek help for their friends than for themselves [18,19].

**Table 6:** Means, SDs, and frequency distributions of responses on perceived usability, perceived usefulness, and intention to use the app (part 2 of the Technology Acceptance Model–based questionnaire; N=38).

		Value, mean (SD)	1: Completely disagree, n (%)	2: Disagree, n (%)	3: Neutral, n (%)	4: Agree, n (%)	5: Strongly agree, n (%)
**Perceived usability**							
	Do you find the app easy to use?	4.29 (0.60)	0 (0)	0 (0)	3 (8)	21 (55)	14 (37)
**Perceived usefulness**							
	Young people might benefit from this app	4.26 (0.50)	0 (0)	0 (0)	1 (3)	26 (68)	11 (29)
	This app will raise awareness of the risks of drug use, both physical and mental	4.23 (0.67)	0 (0)	1 (3)	2 (5)	22 (58)	13 (34)
	The app will provide tools to assess levels of personal risk from drug use	4.10 (0.79)	0 (0)	1 (3)	7 (18)	17 (45)	13 (34)
	The app will help identify red flags that require immediate attention	4.50 (0.60)	0 (0)	0 (0)	2 (5)	15 (40)	21 (55)
	The app will prevent drug use or encourage safe consumption	3.60 (0.85)	0 (0)	3 (8)	15 (40)	14 (37)	6 (16)
	The app will *harm* young people by encouraging them to use drugs (low score means high benefit)	1.89 (0.86)	14 (37)	16 (42)	6 (16)	2 (5)	0 (0)
**Intention to use the app**							
	I would download the app and use it for my personal needs	3.58 (0.98)	0 (0)	6 (16)	12 (32)	12 (32)	8 (21)
	I would download the app and use it to help others	4.21 (0.80)	0 (0)	2 (6)	2 (6)	16 (49)	13 (39)
	I would recommend the app to a friend	4.29 (0.60)	0 (0)	0 (0)	3 (8)	21 (55)	14 (37)

### Study 3: Expert Interviews

We interviewed 10 professionals: 2 (20%) in the area of resilience development, 2 (20%) in the area of substance abuse prevention, 2 (20%) in the area of early intervention to prevent drug-induced mental crises, 1 (10%) rehabilitation and treatment expert, and 3 (30%) search and rescue professionals. Table 7 presents a high-level summary of the experts’ opinions regarding whether the SafeGuard app should include the 5 high-level features (objective 3), along with ideas about how to implement them, including knowledge sources (objective 4), which are presented as footnotes below the table. Multimedia Appendix 2 [46-50] presents specific suggestions for improving the presentation of the app’s features and sources of information other than those listed here.

**Table 7:** High-level summary of opinions of experts regarding the proposed app (N=10).

Feature		Experts, n (%)
**The idea of the SafeGuard app**		
	In favor	10 (100)
	Against	0 (0)
	Neutral	0 (0)
**Risk assessment^a^**		
	In favor	4 (40)
	Against	2 (20)
	Neutral	4 (40)
**Self-monitoring^b^**		
	In favor	4 (40)
	Against	0 (0)
	Neutral	6 (60)
**Useful information^c^**		
	In favor	8 (80)
	Against	0 (0)
	Neutral	2 (2)
**Resilience building^d^**		
	In favor	8 (80)
	Against	0 (0)
	Neutral	2 (20)
**Support center^e^**		
	In favor	9 (90)
	Against	0 (0)
	Neutral	1 (10)

^a^Risk-assessment questionnaire [60].

^b^Ad hoc guiding questions.

^c^Web-based library of the Israel Anti-Drug Authority [61].

^d^Specific methods mentioned by the experts include guided imagery, breathing exercises, movement exercises, mindfulness exercises, focusing on others when distressed, self-compassion development, and criticism reduction.

^e^Descriptions of symptoms that should raise red flags [62,63]; warnings from the Israel National Security Council [64].

## Discussion

### Overview

Our findings suggest that young people would indeed use an app designed to deliver personalized information on the effects of drug use, calculate one’s personal risk for a mental breakdown, and recommend ways to deal with challenges and pressures in ways that increase mental resilience. The findings also support the 5 higher-order requirements and most of the specific features we envisioned for the app, as well as introducing 2 new features that might be incorporated. All the findings point to the app as being particularly useful in the context of long-term backpacking trips—a common rite of passage in which young adults spend time exploring their identity and trying new experiences in distant and unfamiliar locales, away from family and other supportive adults (eg, teachers).

In the sections that follow, we discuss the findings in relation to previous works in the literature, note the study’s strengths and limitations, and offer directions for future research.

### Principal Findings

#### Feasibility

Our participants expressed moderate to high interest in using the app themselves and high interest in using the app to help their friends. These findings are consistent with findings in the literature, which indicate that young people are often more comfortable seeking help for their peers than for themselves [17,18,65]. Although the possibility of accessing support through an app rather than through public services may remove some of the many barriers to seeking help among young people, a certain reluctance to accept that they themselves might need such services still remains. However, the relatively high interest reported by our participants overall is encouraging.

Interestingly, the participants proposed a new user persona that we did not think of ourselves but which we find valuable: the parents or family members of a young person who might be at risk. Rickwood et al [17] argue that interventions should be targeted at the individuals to whom young people turn for information and help. Indeed, interventions by people in a young person’s environment—parents, siblings, friends, and others—may help prevent mental breakdowns, stop mental disorders from worsening, and reduce distress [66,67]. The individuals who interact with a young person at risk are usually aware that they are well positioned to help, but they do not always have the tools, knowledge, and ability to do so [68]. Our proposed app can provide the supportive individuals with the requisite tools and knowledge to intervene at different stages of the young person’s experience (eg, before, during, or after experimentation with recreational drugs).

Finally, we asked the participants to share with us the frequency with which they expected to use the app. This was important not only in terms of assessing interest in the app, but also in terms of our ability to improve the app later on by means of user data. For example, data on the effects of resilience-building exercises from people using the app on a daily basis and willing to share certain nonidentifying personal details (eg, changes in mood) could feed a machine learning algorithm, which could allow the app to predict which types of resilience-building activities are most helpful for different types of users. Similarly, we hoped to be able to discover new risk factors by automatically analyzing structured questionnaire replies and free-text reports. In fact, none of the participants expressed an intention to use the app daily, and only 16% (6/38) of the participants said that they would use the app often. Although we were disappointed in this finding, it was valuable for us to know the limits of the app’s envisioned prediction features.

#### Design of the App

As described previously, we organized the app’s features into five categories: risk assessment, self-monitoring, useful information, resilience development, and a support center. The findings of the study provided encouraging support for these categories. Through the discussion of the mock-up prototype and the written questionnaire in study 2, the focus group participants volunteered functional requirements and additional features that were organized around these categories. In addition, the participants contributed the nonfunctional requirement of anonymity, consistent with extensive literature [20,69-71]. The participants objected to the photo-upload feature in our mock-up, an objection consistent with the desire for anonymity. In this vein, some participants saw value in sharing written testimonials that could help others while not exposing their identity, in line with the study by Schueller et al [72]. However, we do not know whether all participants understood that to be perceived as credible, app contents (such as tips or advice) should disclose the authority of the author, representing the level of expertise of the person or persons writing the information, as well as information regarding the objectivity of the information (ie, how impartial and unbiased the source is) [73]. It is likely that not all participants were conscious of the conflict between privacy and credibility when sharing data with others and the fact that increasing credibility (by disclosing a name or even a nickname) could compromise privacy.

The participants also mentioned an ability to communicate with a counselor for support or help (Figure 1: “Support Center: Anonymous counseling chat”) [26,74] as a desired feature. In fact, our proposed app offers several ways to receive instant support from real people (through the support center) as well as useful information. Our focus group participants also concurred with previous findings [33,75] that the amount of detailed information presented in mental health apps must be balanced with the need for a simple design, which enhances the user experience and promotes engagement.

Among the main features in our proposed app is CBT-based resilience-building activities, along with mindfulness exercises, goal setting, journaling experiences and feelings, and activities for promoting social connections, which is encouraged in the literature [24,76]. Several participants in the focus groups suggested that we incorporate reminders to engage with the app’s situational risk–assessment and resilience-building features, a requirement that is congruent with the literature [77] and that we readily adopt.

Concerning objectives objective 3 and objective 4, all the experts we interviewed appreciated the potential usefulness of the proposed SafeGuard app. All the experts agreed on the importance of resilience development and the support center. Most of the experts agreed that self-monitoring, providing useful information, and risk assessment are useful features. The experts were able to provide useful knowledge sources for risk assessment, useful information, resilience building, and the support center. For self-monitoring, they suggested ad hoc guiding questions.

### Strengths, Limitations, and Future Directions

#### Strengths

An important strength of this study is its use of both quantitative (questionnaires) and qualitative data to identify the requirements for the proposed app. This multi-method approach strengthens the reliability and validity of the findings. Furthermore, engaging end users throughout the development and evaluation processes enables a deep form of participation and a better understanding of patients’ diverse health needs, as well as supports focusing on, and responding to, what matters to patients [78]. This has been advocated and reflected in regulations such as England’s Patient and Public Participation Policy [78]. A related strength of our study relates to the raw nature of the design and the contents of the various features that were presented to the participants. The raw design was intentional because we wanted to gather potential users’ opinions before investing time in a more precise design and to minimize potential bias due to too much detail. Consequently, it is impossible to rule out that a certain portion of the variance in the answers stems from different interpretations of the same features.

#### Limitations

However, our methods included some significant limitations that call for caution in interpreting the results. First, unlike common participatory design processes, the 5 key high-level features of the app were identified without any participant involvement. However, they were based on insights drawn from existing behavioral theories and on a review of similar apps in the literature and in the market. Hence, these features are not arbitrary.

Second, anticipated end users were included in the study; however, their views do not represent the views of all anticipated end users. Although we collected data from young people around the country, we derived our findings based on a limited sample size that overrepresents young secular Jewish female students (it should be noted that overrepresentation of female students is a common finding in web-based mental health research) [79]. Finally, another limitation of this study relates to its exclusive reliance on reporting intentions to use the app, rather than actual behavioral data. This limitation will be addressed in future studies (see the next section).

#### Future Directions

Future research should address fundamental questions about behavior change regarding drug experiences to broaden understanding regarding the motivations, self-efficacy, and triggers [80] that drive healthy young people to become more engaged with mental health apps and especially to use resilience-building activities to decrease the risk of a potential crisis after drug use.

Future research should also examine the relevance of the proposed app in light of the COVID-19 pandemic. The pandemic has changed many aspects of daily life, affecting people’s behavior, lifestyle, and sense of security [81-85]. Young people facing early adulthood during the pandemic have had to cope with increased feelings of uncertainty and anxiety. Studies around the world show that the pandemic has led to increased consumption of drugs, especially cannabis [82-85]. Our proposed app, which offers ways for young people to self-assess their own risk and develop resilience, has the potential to be helpful during the present collective crisis.

Once a beta version of the app is developed, usability and marketing testing studies will be conducted using a small group of real users who will be using the app for several months. The usability testing study will collect the beta testers’ feedback regarding their overall impression of the app; the degree to which it meets their needs; and whether the app’s UI, design, and features are all deemed necessary. The marketing testing study will collect the beta testers’ ideas and feedback on ways to attract attention and distribute the app. Among the key issues we intend to test in this study are (1) the type of messages to use to advertise the app (*mental health safeguard*, *resilience builder*, etc), (2) where to advertise it in the real world (eg, travelers’ clinics, backpackers’ equipment stores, Vipassana retreats, and trance music festivals) and on the internet (eg, specialized websites and communities of travelers), and (3) which social influencers to use. Once the final version is launched, we will test the degree to which it achieves its goals using a large-scale effectiveness study with a randomized controlled design.

### Conclusions

Although they are exploratory in nature, these findings provide preliminary compelling support for the feasibility of the proposed SafeGuard mHealth app. In addition, they provide important insights and information regarding the features that young people, who are the target audience for the app, and experts in the field view as important. Some of these insights (eg, the one related to allowing an option to use the app for a friend) may be useful for the development of other mHealth apps.

## Appendix

Multimedia Appendix 1 Evidence trace table: contexts of use and characteristics of potential users.

Multimedia Appendix 2 Expert evaluations and feedback.

## Abbreviations

ANOVA: analysis of variance
CBT: cognitive behavioral therapy
IDEAS: Integrate, Design, Assess, and Share
mHealth: mobile health
UI: user interface
