# Supplementary material for: Participatory Design of a Mobile App to Safeguard Mental Resilience in the Context of Drug Use in Young Adults: Multi-Method Study
Source: JMIR Form Res. 2022 Feb 25;6(2):e34477. doi: 10.2196/34477 (PMC8917440; doi:10.2196/34477)
Supplement: Multimedia Appendix 1 [file formative_v6i2e34477_app1.docx]

Multimedia Appendix 1. Evidence trace table: contexts of use and characteristics of potential users.

| Sample Supporting Quotes from Study 2 (Hypothetical Personas) | Code | Theme |
| --- | --- | --- |
| *"A 26-year-old girl who was previously diagnosed with a mental disorder and is going on a trip alone for the first time. Wants to keep her 'eyes open' to be sure that she maintains her mental health".* | Before an experience | WHEN |
| *"After using other drugs when thinking over whether to use another drug. Maybe people will open the app to read about it."* |  |  |
| *"It does not seem to me that young people will use it before experimenting with drugs. Only afterwards, or at a time of distress."* | After an experience |  |
| *"On a trip abroad, most of the young people are fearless. You see everyone doing things and everything is fine… They will think the app is exaggerating because they see* *in practice, that everyone is fine… They will use the app in a moment of distress, after use, or while experiencing something strange."* |  |  |
| *"Someone who returns from a long trip abroad and feels a kind of gap between what he experienced and the returning… so needs help getting back to a routine."* |  |  |
| *"A 27-year-old man started using soft drugs, not daily. Following a relationship that made life difficult for him and additional financial problems, he increased his drug use. His condition overcame him, and he broke up with his girlfriend. He collapsed, was unable to function, did not eat, completely lost his career. He sees that his condition is not normal. Before he goes to a psychologist, he wants to use the app to treat himself."* | In times of crisis or stress |  |
| *"A young man who flew to Thailand after ending a relationship is in crisis. He is exposed to drug experiences and sexual experiences. He is interested in finding someone to guide him through a period of search and identity crisis, as well as the things he wants to experience."* |  |  |
| *"The app cannot prevent [problems]… but may be able to direct where to turn to in time of need."* |  |  |
| *"It can help in moments of crisis, when someone loses sight of things, both on drug issues and in general."* |  |  |
| *"A young man who has just been discharged from the army wants to experiment but is afraid."* | Post-military service |  |
| *"A girl, after discharge from the army, traveled with a friend to India. She feels that her friend is overdoing it and smoking marijuana too much…"* | On a trip abroad |  |
| *“A 32-year-old guy being worn down by routine who increases his drug use, has a child, and is afraid of experiencing a crisis.”* | As part of the daily routine |  |
| *"A 25-year-old girl goes out a lot and uses soft drugs to pass the time. She mostly smokes marijuana and does not seem to enjoy life without it. Her friend cares for her and wants to help her."* |  |  |
| *"A young man, 23 years old, in the middle of a trip to India who feels that something is happening to him that he has not experienced before. He wants to use the app to understand what he is going through."* | Young people themselves | WHO: POTENTIAL USERS |
| *"A young woman, diagnosed with fibromyalgia, who wants to try medical cannabis and is unable to get approval. Wants to know what the difference is between ‘regular marijuana’ and the medicinal kind because she is desperate and wants to know how to do it correctly and safely."* |  |  |
| *"I think there is a population that will use such an app, more curious or apprehensive people."* |  |  |
| *"If a person had experienced mental health challenges or mental illness in their family, he would want to have his 'eyes open' while making a decision like traveling alone or trying drugs or spiritual experiences.”* | Young people with prior exposure to mental health problems |  |
| *"The parents of a young man who wants to know how to engage in a conversation with their son regarding drug use and unsafe behavior."* | Parents and family members |  |
| *"A young man who goes to work abroad. His parents feel that he sounds different when they speak to him. They worry. They would like the app to advise them on what questions to ask him and what answers to expect to understand his condition."* |  |  |
| *"People do not tend to think they have a problem and therefore will not download an app [for their own use]. Most of them will download it to help friends."* | Friends |  |
| *"A 23-year-old woman is traveling in Australia while her brother at home is in a difficult mental state. She is barely in touch with her family but is in touch with a good friend of hers. He thinks it would be good if she had a way to get information in moments of crisis or difficulty and wonders how he could help her with that.”* |  |  |
| *"A 22-year-old friend who was discharged from the military and went on a post-military trip to South America. He wants to be able to detect worrying symptoms in his friends. He is more aware and sensitive to the situation. He is the person in charge."* |  |  |
