# Supplementary material for: Participatory Design of a Mobile App to Safeguard Mental Resilience in the Context of Drug Use in Young Adults: Multi-Method Study
Source: JMIR Form Res. 2022 Feb 25;6(2):e34477. doi: 10.2196/34477 (PMC8917440; doi:10.2196/34477)
Supplement: Multimedia Appendix 2 [file formative_v6i2e34477_app2.docx]

Multimedia Appendix 2. Expert evaluations and feedback.

| Feature | Experts in favor | Comments & suggestions |
| --- | --- | --- |
| The idea of the SafeGuard app | 10 of 10 (100%) | H3:   - 6 experts thought the app should provide guidance in case of an emergency - 4 experts thought it should provide information about drugs - 4 experts thought it should make harm-reduction methods accessible for the users - 2 experts thought it should provide legal information about drugs |
| Risk assessment | 4 of 10 (40%)  2 against  4 neutral | H3:   - 3 experts recommended changing the name of the risk-assessment questionnaires to be more understandable and less intimidating, for example "Assess your wellbeing" or "Assess your mental state." - Experts’ concerns about assessing the risk regarded 1) response bias in questionnaires, 2) making the user feel a false sense of confidence, thus being less careful and that 3) the personalized feedback would affect the risk height. - Provide a disclaimer about the accuracy of the questionnaire. Highlight that the intention of the app is to lower risk and build resilience - Provide general or personally adjusted information about risk factors instead of a risk assessment. - Visualization of risk score via red and black flags: Perhaps replace with a wider color scale or use the traffic light analogy (without a green light). - All feedbacks should include talking to a friend, eating and sleeping well, self-hygiene, performing routine daily activities. - Feedback should highlight problematic situations. - Provide advice about how often and in what situations to reassess risk and set reminders (according to a user's preference). - One expert was in favor of risk assessment but suggested that instead of the self-evaluation, a friend of the user should answer 3 questions about the user: previous trauma, abnormal behavior, drug use.   H4: One of the experts recommended using [46] for risk assessment. |
| Self-monitoring | 4 of 10 (40%)  6 neutral | H3:   - 4 experts agreed with using a self-reported question, e.g. "How do I feel?", answered on a sliding scale as in Figure 1, or via emojis and their textual descriptions. - Provide an option to record speech that would be transcribed into writing - The terminology of the directed questions shouldn’t be professional   H4: 4 experts recommended adding directed questions, such as “what helped me today?” “Where was I?” “Whom did I meet?” “What mental state was I in?” “What thoughts helped me today?” “What thoughts were counterproductive to me today?”. |
| Useful information | 8 of 10 (80%)  2 neutral | H3:   - 2 experts emphasized that the information provided should be straightforward, precise, appropriate for the user’s needs, and not overwhelming or frightening. One also advised including information about the advantages of drug use as well as the risks, primarily the short-term consequences. - 5 experts suggested giving only general information about mental distress symptoms, emotional vulnerability when traveling and triggers for mental distress. 2 suggested to explain about symptoms of anxiety, depression, and psychosis but others were concerned it will lead users to over diagnosing. - 2 experts suggested adding legal information about drugs in different countries. - 1 expert thought it’s important to explain about the risk in combining spiritual experiences and drugs. - Experts pointed out the difficulty of knowing the exact kind of drug (or combination of drugs) someone has consumed, and the wide range of reactions it could lead to. Therefore, giving accurate information regarding drug combinations would be unrealistic and could be problematic, even though the demand and need for this information should be high. - One expert didn’t think that the app should include drug-specific information and instead should present information about risk factors for mental crises and important symptoms to monitor.   H4: One expert suggested providing links to harm-reduction sites. Another recommended citing information from the online library of the Israel Anti-Drug Authority [47]. |
| Resilience building | 8 of 10 (80%)  2 neutral | H3:   - 2 experts suggested to change the feature’s name, for example “personal development”. - Social media use should be kept moderate. - Goal setting. One expert suggested a closed set of options such as new experiences, independence, new cultures, clearing one’s head, with an “other” option. Another possibility is directed questions, e.g., “When I return home what experiences would I like to have?” “What was really important for me when I went on this trip?” “If a caring adult was with me, what would he wish for me?”   H4:   - Mindfulness. One expert suggested guided imagery methods rather than unguided mindfulness to prevent excessive rumination. - A resilience-building expert suggested breathing exercises, movement exercises and mindfulness exercises, along with focusing on others when distressed, self-compassion development, and criticism reduction. |
| Support center | 9 of 10 (90%)  1 neutral | H3:   - Chat with an expert is important. - Emergency contact numbers are important and have to be constantly updated. - List of symptoms of mental crises and guidelines to help a friend.   H4:   - Telephone numbers for support hotlines, including Israeli embassies, the Harmony treatment and rehabilitation village (Kfar Izun), Magnus International Search and Rescue, and Chabad House Jewish Community Centers. - Two experts recommended specific sources for descriptions of alarming symptoms [48, 49]. - One expert suggested a link to warnings for travelers by the Israel National Security Council [50]. |
